# Differential Effects of Wild Blueberry (Poly)Phenol Metabolites in Modulating Lipid Metabolism and Oxidative Stress in 3T3‐L1 Adipocytes

**DOI:** 10.1002/mnfr.70101

**Published:** 2025-05-09

**Authors:** Samuele Venturi, Marco Rendine, Mirko Marino, Dorothy Klimis‐Zacas, Patrizia Riso, Cristian Del Bo'

**Affiliations:** ^1^ Division of Human Nutrition Department of Food Environmental and Nutritional Sciences (DeFENS) Università degli Studi di Milano Milan Italy; ^2^ School of Food and Agriculture University of Maine Orono Maine USA

**Keywords:** 3T3‐L1 adipocytes, blueberry, lipid accumulation, oxidative stress, polyphenols

## Abstract

Adipocyte hypertrophy, driven by lipid accumulation, is crucial in the development of obesity. Wild blueberry (WB; *Vaccinium angustifolium*) (poly)phenols (PPs) metabolites may modulate adipogenesis and the development of obesity. This study examines WB PP metabolites’ effects on lipid accumulation, lipid metabolism, and oxidative stress in mature 3T3‐L1 adipocytes. Differentiated 3T3‐L1 adipocytes were treated for 48 h with free fatty acids (FFAs; oleic/palmitic acid 750 µM, 2:1 ratio) and WB‐derived PPs, including ferulic acid (FA), isoferulic acid (IA), vanillic acid (VA), and syringic acid (SA) at physiological and supra‐physiological concentrations. Assessments included lipid accumulation, glycerol release, and markers of lipid metabolism (sterol regulatory element‐binding protein 1c [SREBP‐1], fatty acid synthase [FASN], FAB4) and oxidative stress (DNA damage, 8‐hydroxy 2‐deoxyguanosine [8OHdG], nuclear erythroid factor 2‐related factors 2 (NRF2), heme oxygenase 1 [HO‐1]). FFAs significantly increased lipid accumulation, glycerol release, and FASN levels, while reducing HO‐1 levels, without affecting other markers. WB PP metabolites did not reduce lipid accumulation, but IA and VA reduced FASN levels (−25% and −26%; *p* < 0.05), and SA improved HO‐1 levels (+150%; *p* < 0.05). Despite the different effects observed, the findings obtained under our experimental conditions seem to suggest that IA, VA, and SA may modulate lipid metabolism and oxidative stress markers. However, further studies are fundamental to corroborate the findings obtained and support the contribution of these BB PPs metabolites and other compounds in the prevention and management of obesity.

## Graphical abstract

Metabolites derived from wild blueberry (poly)phenols, such as syringic (SA), vanillic (VA), and isoferulic acids (IA), can modulate lipid metabolism and oxidative stress markers in mature 3T3‐L1 adipocytes treated with free fatty acids (FFAs; oleic/palmitic acid, 750 µM, 2:1 ratio). Our findings demonstrate that VA and IA enhance FASN levels, a key marker of lipid metabolism disrupted by FFAs, while syringic acid restores the expression of the antioxidant enzyme HO‐1, inhibited by FFAs.

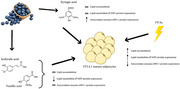

**Abbreviations**
8OhdG: 8‐hydroxy 2‐deoxyguanosine
ACN: anthocyanin
FA: ferulic acid
FABP‐4: fatty acid binding protein 4
FASN: fatty acid synthase
FBS: fetal bovine serum
FFA: free fatty acid
HO‐1: heme oxygenase 1
IA: isoferulic acid
IBMX: 3‐isobuthyl‐1‐methylxanthine
NE: norepinephrine
NRF2: nuclear erythroid factor 2‐related factor 2
OA: oleic acid
PA: palmitic acid
PBS: phosphate‐buffered saline
PP: (poly)phenol
ROS: reactive oxygen species
SA: syringic acid
SREBP‐1c: sterol regulatory element‐binding protein 1c
TX‐100: TritonX100
VA: vanillic acid
WB: wild blueberry

## 1 Introduction

Obesity is a complex metabolic disorder defined by excessive accumulation of body fat, which has become a significant global health concern in recent years. In the context of this epidemic, adipose tissue, particularly adipocytes, plays a crucial role. Adipocytes, commonly referred to as fat cells, are specialized cells responsible for the storage and mobilization of triglycerides [1]. Although the primary function is energy storage, adipocytes also fulfill critical functions in metabolic regulation and hormone secretion, such as adiponectin and leptin, which are involved in energy balance, inflammation, and immune response [2]. Adipose tissue in obesity is characterized by an increase in mature adipocyte size through lipid accumulation (hypertrophy), and in the formation of new adipocytes from preadipocytes, precursor cells at the level of adipose tissue (hyperplasia) [3]. Typically, the quantity of adipocytes in adipose tissue is established early in life, but in the context of energy intake excess, preadipocyte differentiation may contribute to the enlargement of adipose tissue [4]. This expansion resulting from adipogenesis, and lipid accumulation correlates with elevated mechanical stress, leading to hypoxic stress, enhanced inflammatory processes, oxidative stress, and systemic insulin resistance within the adipose tissue [3]. Several mechanisms are involved in lipid accumulation and adipogenesis during chronic overnutrition and insulin resistance. The levels of free fatty acids (FFAs) and triglycerides (TGs) in the blood increase, by promoting the uptake of FFA and their accumulation as TGs in cytoplasmic lipid droplets in mature adipocytes [5]. Furthermore, high lipid levels in adipocytes activate the sterol regulatory element‐binding protein 1c (SREBP‐1c), a transcription factor that increases the expression of peroxisome proliferator‐activated receptor γ (PPAR‐γ), a key factor that regulates adipocyte‐specific proteins, involved in lipogenesis and adipogenesis, including fatty acid synthase (FASN) and fatty acid binding protein 4 (FABP4) [6, 7, 8]. Furthermore, obesity is characterized by an impaired antioxidant capacity with increased production of reactive oxygen species (ROS) and reduced antioxidant defense, which negatively modulates lipid accumulation in adipose tissue [9]. The production of ROS leads to DNA oxidative damage resulting in an increase in catabolites such as 8‐hydroxy 2‐deoxyguanosine (8OHdG) [10]. Moreover, ROS production promotes the activation of nuclear erythroid factor 2‐related factors 2 (Nrf2), a transcriptional factor that binds to antioxidant response elements (AREs), promoting the upregulation of enzymes involved in the prevention of oxidative damage, such as heme oxygenase 1 (HO‐1), superoxide dismutase (SOD), catalase, and glutathione peroxidase [11].

Blueberries, in particular wild blueberries (WBs, *Vaccinium angustifolium*), represent one of the most widely consumed berries. Several studies carried out in our laboratories reported the role potential of WB in the modulation of several metabolic and functional activities both in cell culture [12, 13] and animal models [14, 15, 16], while results from human intervention studies are few and inconsistent [17, 18, 19]. Most of their beneficial effects are attributed to their nutritional composition. In fact, WBs are an important source of dietary fiber, vitamins, minerals, and above all (poly)phenols (PPs), which are predominantly represented by anthocyanins (ACNs) and phenolic acids, followed by flavonols, flavan‐3‐ols, and proanthocyanidins [20, 21]. In particular, the ACNs content in WB ranges from 57 to 503 mg per 100 g of raw fruits [22]. The main ACNs are malvidin, cyanidin, petunidin, delphinidin, peonidin, and pelargonidin, which are linked to glucoside, galactoside, and arabinoside, while the main phenolic acid is chlorogenic acid, which average content ranges from 43 to 114 mg per 100 g of raw fruit [22, 23]. PPs are characterized by a low bioavailability, with approximately 5%–10% of the PPs intake being absorbed at the small intestine level. Only a small portion of the parent compounds (about 1%) reaches the circulatory system unaltered and is subsequently excreted in urine [23, 24]. In fact, the majority of PPs undergo deconjugation reactions (i.e., deglycosylation) before uptake. This process occurs through various mechanisms, including passive diffusion (for low‐weight molecules like phenolic acids, flavonoid aglycones, and catechins), sodium‐glucose transporters like SGLT‐1 (for some glycoside PPs), and facilitated transport [25, 26]. PPs that remain unabsorbed reach the colon, where gut microbiota extensively metabolizes them into smaller molecules that can be absorbed [24, 27, 28]. After enterocyte uptake, PPs and their metabolites undergo further modifications through Phase I (i.e., hydrolysis, reduction, and oxidation) and Phase II (i.e., glucuronidation, sulfatation, and methylation) metabolism. This biotransformation produces water‐soluble conjugated molecules that are transported through the bloodstream to organs, tissues, and cells and are ultimately excreted with urine [25, 29]. Although their concentration reaches picomolar or nanomolar concentrations in the blood, several studies have documented the capacity of WB and their PPs to exert a plethora of biological activities in vivo and in vitro. Specifically, they have been shown to counteract oxidative stress and inflammation, ameliorate glucose and lipid metabolism, and alter gut microbiota composition [18, 30, 31, 32]. In addition, WB PPs have been reported to exert a potential role in the modulation of adipocyte differentiation, lipid accumulation, thus playing a role against metabolic syndrome and obesity [33, 34, 35, 36, 37, 38, 39].

The aim of the present study was to evaluate the effect of WB PPs metabolites on lipid accumulation and lipogenesis in mature 3T3‐L1 adipocytes treated with a mix of FFAs. The compounds tested included ferulic acid (FA), isoferulic acid (IA), vanillic acid (VA), and syringic acid (SA), which are among the main metabolites found in the bloodstream after the consumption of a portion of blueberries [40, 41, 42]. The compounds were tested at physiological and supraphysiological concentrations, alone and in combination. The effect on lipid accumulation was evaluated through the expression of sterol regulatory element binding protein 1c (SREBP1c), FASN, and FABP4 as the main pathways of lipid metabolism, while the effect on oxidative stress was quantified by analyzing the production of Nrf2, HO‐1, and DNA damage. To the best of our knowledge, the involvement of WB polyphenols (PPs) and their metabolites in lipid accumulation and oxidative stress in mature adipocytes remains largely unexplored. In fact, while previous studies have investigated the effects of whole berry extracts or other individual polyphenols (i.e., quercetin, naringenin, chlorogenic acid, and coumaric acid), research specifically focusing on circulating metabolites (FA, IA, VA, and SA) and their role in adipocyte dysmetabolism is lacking. Thus, this is the first study to evaluate the direct effects of these metabolites derived from the consumption of WB on lipid metabolism and oxidative stress in mature adipocytes. Given the increasing recognition of the importance of polyphenol metabolism in determining biological activity, our findings provide a critical step forward in understanding the role of these metabolic products. This work not only fills a significant gap in the literature but also advances the field by incorporating physiologically relevant concentrations, thereby bringing research closer to translational scenarios.

## 2 Experimental Section

### 2.1 Chemicals and Reagents

Standards of FA (CAS No. 537‐98‐4), IA (CAS No. 537‐73‐5), VA (CAS No. 121‐34‐6), SA (CAS No. 530‐57‐4), palmitic acid (PA), oleic acid (OA), bovine serum albumin (BSA), norepinephrine (NE), high‐glucose Dulbecco's Modified Eagle Medium (DMEM), penicillin–streptomycin, trypsin‐EDTA, phosphate‐buffered saline (PBS), insulin, 3‐isobuthyl‐1‐methylxanthine (IBMX), dexamethasone, TritonX100 (TX‐100), trypan blue, thiazolyl blue tetrazolium bromide (MTT, Cat. No. M2128), oil red O (Cat. No. O0625), dimethyl sulfoxide (DMSO), and hydrochloric acid were purchased from Merck (Darmstadt, Germany). Fetal bovine serum (FBS) was purchased from Thermo Fisher Scientific (Waltham, MA, USA). Formalin, 2‐propanol, methanol (MetOH), and ethanol (EtOH) were provided by VWR International srl (Milan, Italy). ProteinSafe Protease Inhibitor Cocktail (100×) (Cat No. DI111‐02) was provided by CliniSciences (Guidonia Montecelio, RM, Italy). Water was obtained from a Milli‐Q apparatus (Millipore, Milford, MA).

### 2.2 Preparation of Wild Blueberry (Poly)Phenol Metabolites and Norepinephrine Stock Solution

**FIGURE 1:**
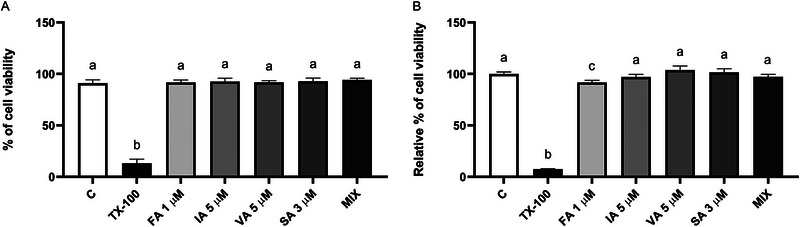
(A, B) Effect of phenolic acids (FA 1 µM; IA 5 µM; VA 5 µM; SA 3 µM; MIX) on cell viability assessed through the trypan blue assay (A) and MTT assay (B). Results are expressed as % and relative % of live cells, respectively. The error bars indicate the standard error of the mean. The significance level was set at 0.05. ^a,b,c^Bar graphs with different letters are significantly different (*p* < 0.05). C, negative control; FA, ferulic acid; IA, isoferulic acid; MIX, FA + IA + VA + SA; SA, syringic acid; TX‐100, Triton‐X 100; VA, vanillic acid.

Lyophilized standards of FA, IA, VA, and SA (Figure 1A–D) were dissolved in MetOH in order to prepare their respective stock solutions at a concentration of 10 mM. The final concentration of MetOH in the medium in contact with cells was 0.05%. Aliquots of the standard were prepared and stored at −20°C until use. An equal concentration of MetOH was applied to the medium of the negative control cells.

NE served as a positive control, capable of counteracting lipid accumulation and inducing lipolysis in mature 3T3‐L1 adipocytes at the concentration of 1 µM [43]. NE stock solution (1 mM) was prepared in an aqueous solution of HCl (10 mM), with a final HCl concentration in the exposure media of 0.0003%.

### 2.3 Preparation of Free Fatty Acids and Control Solution

The stock solution of PA (0.2 M) and FFA mix (0.2 M oleic/PA; 2:1 ratio) was prepared in EtOH and stored at −20°C. Before use, PA and FFAs were dissolved in the cell culture medium solution containing 10% BSA at 37°C [44, 45]. The PA/BSA and FFAs/BSA solutions were added to the medium to achieve final concentrations of 500 and 750 µM for PA or FFAs, respectively. The FFAs combination was chosen because it more accurately simulates in vivo conditions, as both oleic and PAs are prevalent in human plasma [46]. The same concentration of EtOH/BSA was used in the medium of the negative control cells. The final concentrations of BSA and EtOH in the medium were 2% and 0.37%, respectively.

### 2.4 Cell Culture and Adipocyte Differentiation

3T3‐L1 mouse preadipocyte cells (Cat. No. IFO50416), characterized for adipocyte‐like features, were sourced from the Japanese Collection of Research Biosources Cell Bank (JCRB) and purchased from TebuBio (Magenta, MI, Italy). The cells were cultivated in high‐glucose DMEM supplemented with 10% (v/v) heat‐inactivated FBS and antibiotics (50 U/mL penicillin, 50 µg/mL streptomycin) at 37°C in a humidified 5% CO_2_ atmosphere. Subculturing was performed at 80% confluence using 0.05% trypsin/EDTA. Throughout cell growth and differentiation, the medium was refreshed every 2–3 days. Cells within passages 3–10 were utilized.

3T3‐L1 cells were differentiated in mature adipocytes according to the differentiation protocol provided by the American Type Culture Collection (ATCC). Preadipocytes were maintained in a complete DMEM medium for 2 days postconfluence. Differentiation was initiated on Day 0 using a differentiation medium (complete DMEM with 1 µg/mL insulin, 1 µM dexamethasone, and 500 µM IBMX) for 48 h. Subsequently, on Day 2, the culture medium was switched to insulin medium (complete DMEM with 1 µg/mL insulin) and freshly replaced every 2–3 days for the next 5 days. On Day 7, the insulin medium was replaced with a maintenance medium composed of complete DMEM for 48 h. Treatments with FFAs and PPs were added on Day 9 and continued for 48 h until the end of the experiments. 3T3‐L1 cells exhibit the characteristics of mature adipocytes, including the production of intracellular lipid droplets, and achieve full differentiation between Days 7 and 14 from the initiation of the process.

### 2.5 Cell Viability Assay

Cell viability was assessed using the trypan blue exclusion assay and an MTT assay. The trypan blue exclusion assay was performed using a TC20 automated cell counter and dual‐chamber cell counting slides (BIORAD, Segrate, Milan, Italy). 3T3‐L1 cells were seeded in 12‐well plates at a density of 40 000 cells. Upon reaching confluence, cells were treated with PPs at different concentrations either individually (1 µM FA, 5 µM IA, 5 µM VA, and 3 µM SA) or in a MIX (0.1 µM FA, 0.1 µM IA, 0.1 µM VA, and 0.3 µM SA), along with a negative control (C) and the positive control (TX‐100 0.1%), for 48 h. Subsequently, supernatants were collected, cells were detached with trypsin/EDTA (0.05%) and resuspended in the respective supernatants. The medium with cells was used for the trypan blue exclusion assay. Three independent experiments were conducted, with each condition tested in duplicate. Cell viability was considered by measuring the percentage of live cells relative to the total number of cells.

The MTT assay was performed to evaluate cell viability [47]. 3T3‐L1 cells were seeded in 96‐well plates at a density of 5000 cells. After 24 h of culture, cells were treated under the same conditions as those tested in the trypan blue exclusion assay. The treatment medium was replaced by MTT solution (1 mg/mL in complete DMEM; filter sterilized). Following a 4‐h incubation in the dark at 37°C, the MTT solution was removed, and formazan crystals were dissolved in 200 µL of DMSO. The plate was incubated for 20 min at room temperature on a horizontal shaker (300 rpm). Absorbance was measured at 450 nm using a plate reader (mod. F200 Infinite, TECAN, Milan, Italy). Three independent experiments were conducted, with each condition tested in six replicates. Cell viability was calculated as the fold increase in absorbance with respect to the negative control.

### 2.6 Lipid Accumulation by Oil Red O Staining

In order to evaluate the effect of FFAs and PPs in the modulation of lipid accumulation in mature 3T3‐L1 adipocytes, the Oil Red O staining assay was conducted [48].

The potential of FFAs to induce lipid accumulation was evaluated. 3T3‐L1 preadipocytes were seeded in 24‐well plates (20 000 cells/well) and differentiated following the previously described protocol. On Day 9, mature adipocytes were treated with different concentrations of PA (500–750 µM) and FFAs mix (500–750 µM; oleic/PA; 2:1 ratio) for 24 and 48 h. At the end of experiment, the Oil Red O staining assay was performed. Cells were fixed with 10% neutral buffered formalin for 30 min at room temperature, rinsed with PBS twice, and then with 60% 2‐propanol for 5 min. Cells were covered with 0.2% Oil Red O dye at room temperature for 1 h. After three rinses with ultrapure water, 100% 2‐propanol was added, and the plate was gently mixed. Absorbance was read at 550 nm using a plate reader (mod. F200 Infinite, TECAN, Milan, Italy). Three independent experiments were conducted, with each condition tested in four replicates.

After determining the optimal FFA concentration of 750 µM and treatment duration of 48 h for effectively inducing lipid accumulation, PPs were tested. 3T3‐L1 preadipocytes were seeded in 24‐well plates (20 000 cells/well) and differentiated. On Day 9, mature adipocytes were treated with different concentrations of PPs individually (10–100 nM FA, 100–500 nM IA, 100–500 nM VA, and 50–300 nM SA) or in a MIX (100 nM FA, 100 nM IA, 100 nM VA, and 300 nM SA) along with FFAs/BSA (750 µM). Compounds and concentrations were selected based on the results obtained from previous dietary intervention studies in which the bioavailability of PPs from WB was investigated [40, 41, 42]. A negative control was included using DMEM with the vehicle (0.05% MetOH, 0.37% EtOH). The FFAs control consisted of 750 µM FFAs/BSA and 0.05% MetOH. NE at 1 µM served as a positive control capable of reducing lipid accumulation. Cells were treated for 48 h, and then the Oil Red O staining was performed. Three independent experiments were conducted, with each condition tested in four replicates.

### 2.7 Glycerol Release Quantification

Glycerol was detected in the medium using a glycerol assay kit (Cat. No. MAK117; Merck, Darmstadt, Germany), according to the manufacturer's instructions. After adipocyte differentiation on Day 9, 3T3‐L1 cells underwent the same treatment as described above for the Oil Red O staining assay. At the end of the experiments, the culture medium was collected, centrifuged (300 rcf for 10 min), and stored at −80°C until the colorimetric detection of glycerol. For each condition, 10 µL of medium was added in a transparent 96‐well plate, followed by the addition of 100 µL of the master reaction mix. The plate was shaken for 20 min at room temperature in the dark. Subsequently, the absorbance was read at 550 nm using a plate reader (mod. F200 Infinite, TECAN, Milan, Italy). The analysis was conducted in duplicate, and the findings were derived from three independent experiments.

### 2.8 Comet Assay

The evaluation of the effect of the FFAs mix and the phenolic compounds on DNA damage as single‐strand breaks in 3T3‐L1 cells was carried out by the comet assay. 3T3‐L1 cells were seeded at a density of 20 000 cells each well in a 24‐well plate and differentiated as previously described. After the FFAs and phenolic treatments, cells were washed with PBS, detached with trypsin‐EDTA (0.25%), and resuspended in fresh complete DMEM. An amount of 100 000 cells was collected and washed in PBS twice by centrifuging for 10 s at 10 000 × *g*. Finally, cells were used for the comet assay following the procedure used for the mononuclear cells by Marino et al. [49]. Two experiments were performed in duplicate.

### 2.9 Assessment of Lipid Metabolism and Oxidative Stress Markers by Enzyme‐Linked Immunosorbent Assay (ELISA)

3T3‐L1 cells were seeded in a 6‐well plate (80 000 cells/well) in duplicate to ensure an adequate amount of sample for the analysis. After differentiation, mature 3T3‐L1 adipocytes were treated with single PPs at different concentrations (0.1–1 µM FA, 0.5–5 µM IA, 0.5–5 µM VA, and 0.3–3 µM SA) or an MIX (0.1 µM FA, 0.1 µM IA, 0.1 µM VA, and 0.3 µM SA) with FFAs/BSA (750 µM) for 48 h. Negative and FFAs‐positive controls were included. The detection of SREBP‐1C and 8OHdG was performed on the cell culture supernatant, while cell extract was used for the assessment of FASN, FABP4, NRF2, and HO‐1. At the conclusion of the experiments, cell supernatants were collected and centrifuged at 300 rcf for 10 min at 4°C. Protein extraction followed the manufacturer's protocol: cells were detached by trypsin‐EDTA (0.25%), centrifuged at 300 rcf for 5 min at 4°C, washed three times with cold PBS, and resuspended in PBS 1% ProteinSafe Protease Inhibitor Cocktail. Cells were ultrasonicated for 90 s, and vortexed for 15 s four times, subsequently cells were centrifuged at 2000 rcf for 15 min at 4°C, and the supernatants collected. Samples of cell supernatants and cell lysate were stored at −80°C until the analysis. Three independent experiments were conducted in duplicates. ELISA kits were used to detect the protein levels of SREBP‐1c, FASN, FABP‐4, NRF2, HO‐1, and 8OHdG (Cat. No. MBS3807337, MBS 731124, MBS452808, MBS1603021, MBS2702089, MBS269722, respectively; MyBioSource, Inc. San Diego, CA, USA).

### 2.10 Statistical Analysis

Statistical analysis was performed using GraphPad software (GraphPad Software, Boston, MA, USA). Results are expressed as means ± standard error of the mean (SEM). The effects of PPs and FFAs on lipid accumulation, glycerol release, markers of lipid metabolism, and oxidative stress in mature 3T3‐L1 adipocytes were assessed using one‐way analysis of variance (ANOVA). Post‐hoc analysis was performed using the least significant difference (LSD) test, with the level of statistical significance set at *p* ≤ 0.05. The identification and subsequent removal of outliers was carried out through the ROUT test with *Q* = 1%.

## 3 Results

### 3.1 Evaluation of Phenolic Acid Cytotoxicity

The impact of the PPs, tested individually and as a mixture, on cell viability is shown in Figure 1A, B. Overall, the results of both tests are comparable and the phenolic compounds maintained cell viability over 90%. Thus, the exposure to these phenolic acids did not cause apoptosis. Trypan blue and MTT assays were used. Regarding the trypan blue assay (Figure 1A), the treatment with the positive control (TX‐100 0.1%) significantly reduced cell viability compared to the negative control (C; *p* < 0.0001), with a percentage of live cells below 15%. The treatments with FA 1 µM, IA 5 µM, VA 5 µM, SA 3 µM, and the MIX did not decrease cell viability after 48 h (*p* > 0.05). The MTT assay provided similar results (Figure 1B). The positive control significantly decreased the relative percentage of vitality compared to the negative control (C; *p* < 0.0001). The single compounds and the MIX did not induce cell cytotoxicity compared to the negative control (*p* > 0.05), except for the treatment with FA 1 µM that significantly reduced viability (*p* = 0.03), but the relative percentage remained over 90%.

### 3.2 Effect of FFAs on Lipid Accumulation

**FIGURE 2:**
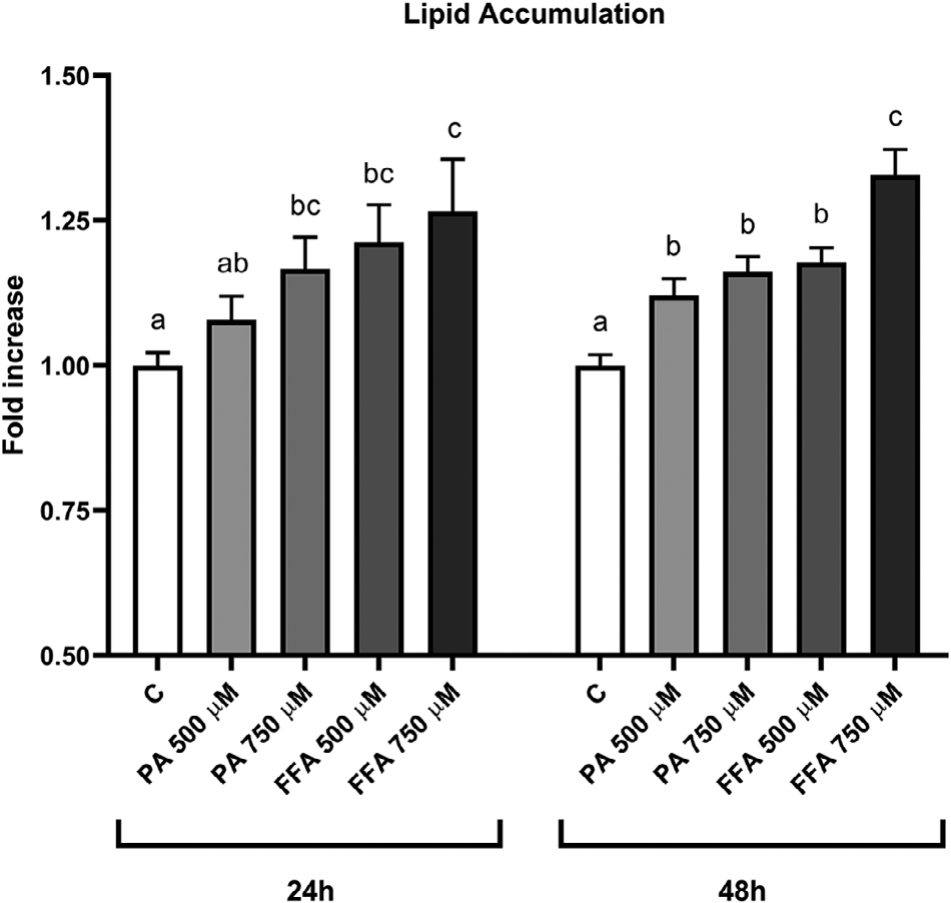
Level of lipid accumulation in mature 3T3‐L1 adipocytes after free fatty acids treatment for 24 and 48 h. Results are expressed as the mean of the fold increases. The error bars indicate the standard error of the mean. FFAs = oleic/palmitic acid; 2:1 ratio. The significance level was set at 0.05. ^a,b,c^Bar graphs with different letters are significantly different (*p* < 0.05). C, negative control; FFA, free fatty acid; PA, palmitic acid.

In order to determine the optimal composition and concentration of FFAs that enhance lipid accumulation in mature 3T3‐L1 adipocytes, the Oil Red O (ORO) assay was conducted. Figure 2 depicts the results of the ORO assay after 24 and 48 h of treatment with PA and FFAs. After 24 h, treatments with PA at 750 µM, FFAs at 500 µM, and FFAs at 750 µM significantly increased lipid storage by 16.7%, 21.2%, and 26.7%, respectively, compared to the control (*p* < 0.05). PA at 500 µM also elevated lipid levels by 7.9%, but not significantly (*p* > 0.05). However, there was no statistical difference between PA at 750 µM, FFAs at 500 µM, and FFAs at 750 µM (*p* > 0.05). Whereas the 48‐h treatment with PA 500 at µM, PA at 750 µM, FFAs at 500 µM, and FFAs at 750 µM significantly increased lipid accumulation in cells by 12.2%, 16.1%, 17.8%, and 32.8%, respectively, compared to the control (*p* < 0.01). In particular, the treatment with FFAs at 750 µM showed a significantly higher effect than the other conditions (*p* < 0.05).

### 3.3 Effect of (Poly)Phenols on FFAs‐Induced Lipid Accumulation

**FIGURE 3:**
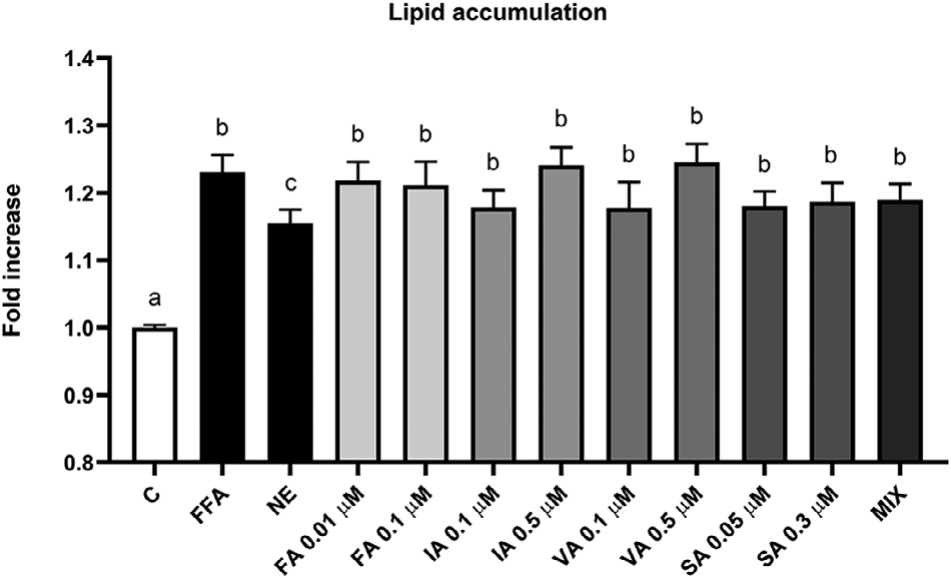
Effect of PPs on lipid accumulation FFAs‐induced in mature 3T3‐L1 adipocytes after 48 h. Results are expressed as the mean of fold increases. The error bars indicate the standard error of the mean. FFAs = oleic/palmitic acid; 2:1 ratio. The significance level was set at 0.05. ^a,b,c^Bar graphs with different letters are significantly different (*p* < 0.05). C, negative control; FA, ferulic acid; FFA, free fatty acid; IA, isoferulic acid; MIX, FA + IA + VA + SA; NE, norepinephrine; PP, (poly)phenol; SA, syringic acid; VA, vanillic acid.

Figure 3 illustrates the potential role of blueberry PPs metabolites in reducing the lipid accumulation in mature 3T3‐L1 adipocytes, as assessed through the ORO assay. The findings reveal that treatment with the FFAs mixture induced a significant increase in lipid accumulation (+23.1%; *p *< 0.0001) compared to the negative control. The use of NE as a positive control showed a reduction in lipid accumulation compared to the FFAs positive control (−6.2%; *p* < 0.05), but it did not reach the level observed in the negative control (*p* < 0.0001). The incubation with the single PPs or the MIX did not significantly alter the storage of lipid droplets compared to the FFAs control (*p* > 0.05).

### 3.4 Effect of (Poly)Phenols on Markers of Lipolysis

**FIGURE 4:**
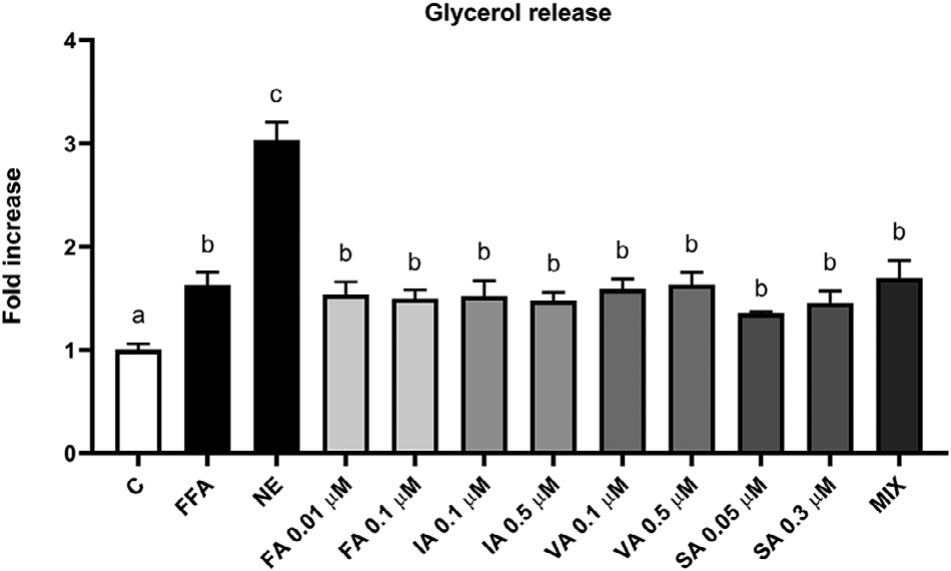
Effect of FFAs and PPs on glycerol release in mature 3T3‐L1 adipocytes after 48 h. Results are expressed as the mean of the fold increases. The error bars indicate the standard error of the mean. FFAs = oleic/palmitic acid; 2:1 ratio. The significance level was set at 0.05. ^a,b,c^Bar graphs with different letters are significantly different (*p* < 0.05). C, negative control; FA, ferulic acid; FFA, free fatty acid; IA, isoferulic acid; MIX, FA + IA + VA + SA; NE, norepinephrine; PP, (poly)phenol; SA, syringic acid; VA, vanillic acid.

In Figure 4, the findings of the effects of PPs on FFAs and glycerol, serving as a marker of lipolysis, are reported. Notably, FFAs appear to induce glycerol release in every condition, compared to the negative control (*p* < 0.001). Treatment with NE markedly induced the release of glycerol compared to the other conditions (*p* < 0.0001).

### 3.5 Effects of (Poly)Phenols on Marker of Lipid Metabolism

**FIGURE 5:**
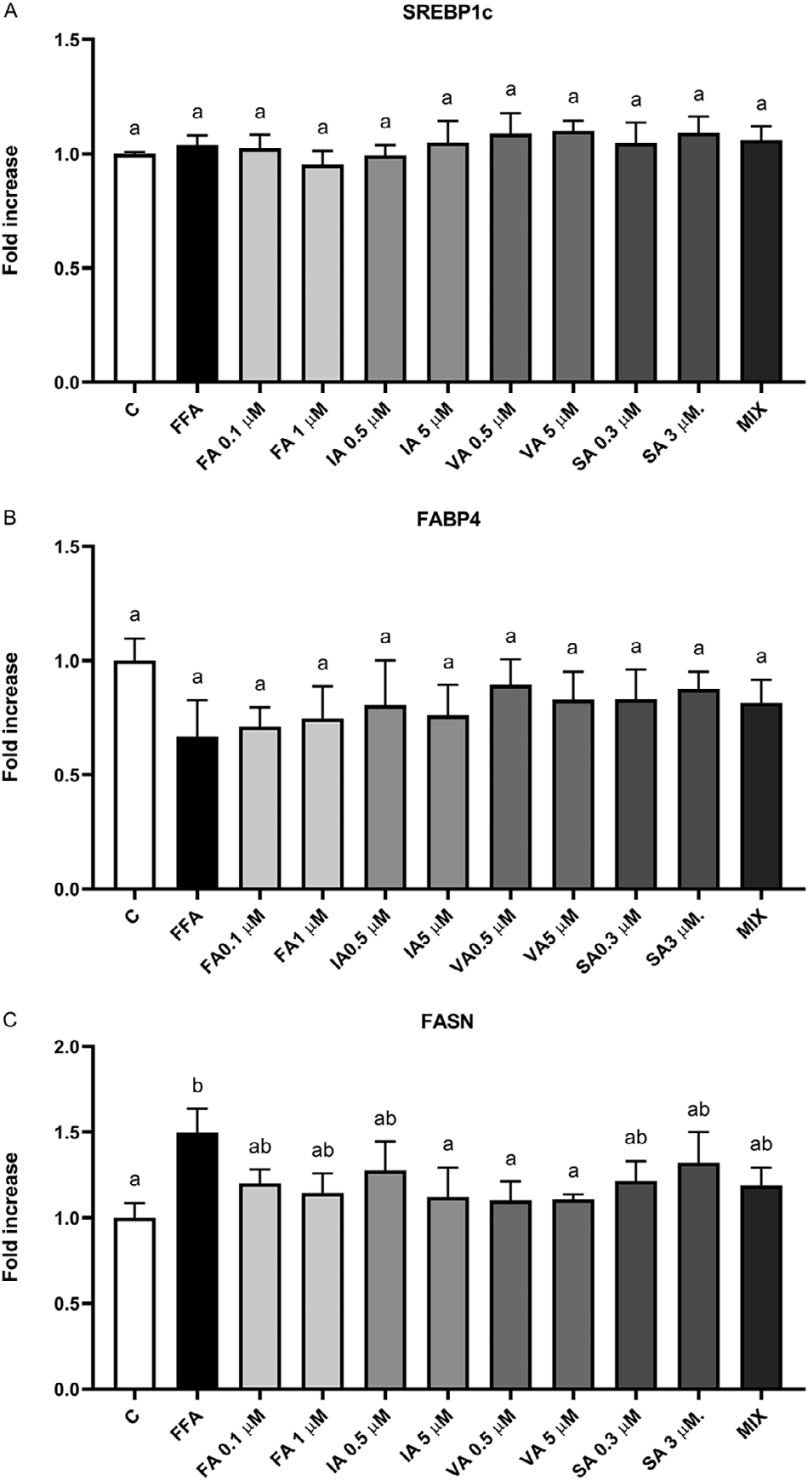
(A–C) Effect of FFAs and PPs on SREBP1c (A), FASN (B), and FABP4 (C) in mature 3T3‐L1 adipocytes after 48 h. Results are expressed as the mean of the fold increases. The error bars indicate the standard error of the mean. FFAs = oleic/palmitic acid; 2:1 ratio. The significance level was set at 0.05. ^a,b^Bar graphs with different letters are significantly different (*p* < 0.05). C, negative control; FA, ferulic acid; FABP4, fatty acid binding protein 4; FASN, fatty acid synthase; FFA, free fatty acid; IA, isoferulic acid; MIX, FA + IA + VA + SA; PP, (poly)phenol; SA, syringic acid; SREBP1c, sterol regulatory element binding protein 1c; VA, vanillic acid.

Figure 5A–C show the effect of the FFAs mix and PPs on markers related to lipid metabolism. The analysis of the cellular levels of SREBP1c and FABP4 revealed no significant effect of FFAs after 48 h of treatment compared to the negative control (*p* > 0.05). Furthermore, treatments with PPs at any concentration did not affect SREBP1c and FABP4 modulation compared to the negative control (p>0.05) (Figure 5A, B).

However, the findings from the analysis of FASN showed a significant increase in the level of FASN in cells treated with only FFAs compared to the negative control (*p* < 0.01) (Figure 5C). In particular, cells treated with IA at 5 µM and both concentrations of VA (0.5–5 µM) exhibited reduced expression of FASN compared to the FFAs positive control (*p* < 0.05), remaining at the level of the negative control (*p* > 0.05). The other treatments showed no statistical difference compared to either C or FFAs treatment (*p* > 0.05).

### 3.6 Effect of FFAs and (Poly)Phenols on Markers of Oxidative Stress

**FIGURE 6:**
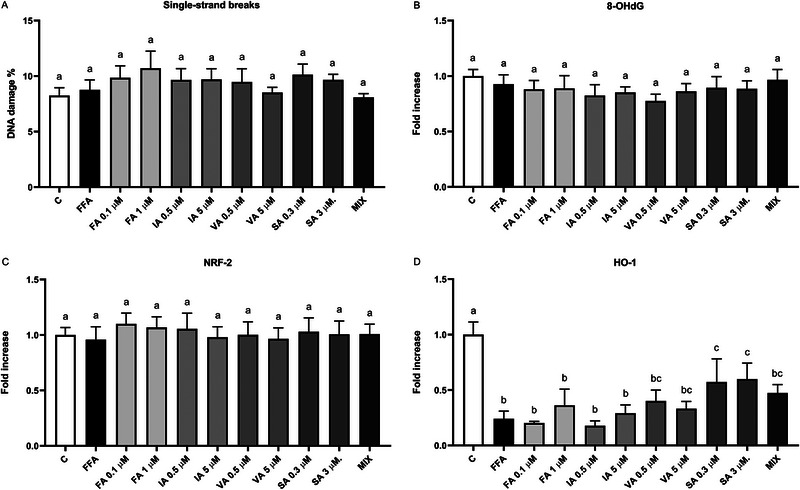
(A–D) Effect of FFAs and PPs on single strand breaks (A), 8OHdG (B), NRF2 (C), and HO‐1 (D) in mature 3T3‐L1 adipocytes after 48 h. Results of single strand breaks are expressed as the mean of the percentage of DNA damage, while the levels of 8OHdG, NRF2, and HO‐1 are expressed as the mean of the fold increases. The error bars indicate the standard error of the mean. FFAs = oleic/palmitic acid; 2:1 ratio. The significance level was set at 0.05. ^a,b,c^Bar graphs with different letters are significantly different (*p* < 0.05). 8OHdG, 8‐hydroxy 2‐deoxyguanosine; C, negative control; FA, ferulic acid; FFA, free fatty acid; HO‐1, heme oxygenase 1; IA, isoferulic acid; MIX, FA + IA + VA + SA; NRF2, nuclear erythroid factor 2‐related factors 2; PP, (poly)phenol; SA, syringic acid; VA, vanillic acid.

The effect of the FFAs and PPs on oxidative stress markers related to DNA damage and the antioxidant pathway in mature 3T3‐L1 adipocytes is depicted in Figure 6A–D.

DNA damage was evaluated as single‐strand breaks using the comet assay and as the release of 8‐hydroxy‐2‐deoxyguanosine (8OHdG). Cells, treated with C, FFAs, and PPs at all concentrations, exhibited a percentage of DNA damage ranging between 8.1% and 10.7% (Figure 6A). Notably, FFAs and PPs treatment showed no modulation of DNA damage in mature 3T3‐L1 cells after 48 h compared to the negative control (*p* > 0.05). Moreover, 8OHdG release in the cell supernatants following both FFAs‐only and FFAs with PPs treatments for 48 h remained unaltered compared to the negative control (*p* > 0.05) (Figure 6B).

Additionally, the level of the transcription factor associated with the antioxidant defense, nuclear factor erythroid 2‐related factor 2 (NRF2) in mature 3T3‐L1 was not affected by FFAs compared to the negative control (*p* > 0.05) (Figure 6C), and PPs did not affect the production of NRF2 either (*p* > 0.05). Figure 6D represents the impact of FFAs and PPs on the production of HO‐1. After 48 h, FFAs significantly decreased the level of HO‐1 compared to the negative control (*p* < 0.001). However, the treatment with both concentrations of SA (0.3–3 µM) ameliorated the FFAs‐induced reduction of HO‐1 significantly compared to the FFAs positive control (*p* < 0.05), although it did not reach the level of the negative control (*p* < 0.05).

## 4 Discussion

In this study, we assessed for the first time the role of WB PP‐metabolites to counteract lipid accumulation and oxidative stress induced by FFAs in mature 3T3‐L1 adipocytes. Overall, we documented that physiological dose of IA and VA, among the main PP metabolites from WB, reduced FFAs‐induced levels of FASN, while no significant effect was observed on lipid accumulation, glycerol release, and other markers of lipid metabolism, including SREBP‐1c and FABP‐4. Regarding oxidative stress, SA was able to mitigate the FFAs‐induced reduction of HO‐1, while no effect was observed for the levels of DNA damage, 8OHdG, and NRF2.

The effect of WB and related PPs in the modulation of lipid metabolism has been evaluated in numerous in vitro studies with controversial results [50, 51, 52, 53, 54, 55, 56, 57]. Koh et al. (2017) observed a reduction in lipid accumulation in mature 3T3‐L1 adipocytes after the treatment with FA at 25 and 100 µM for 48 h [55]. Jung et al. (2018) reported the suppression of lipid accumulation in mature 3T3‐L1 cells following 48 h treatment with VA at 1 and 10 µM, but not at 0.1 µM [57]. Molonia et al. treated mature 3T3‐L1 cells with cyanidin‐3‐*O*‐glucoside at 5–10 µM for 24 h, followed by exposure to PA (1000 µM), in which they found that cyanidin‐3‐*O*‐glucoside decreased lipid accumulation in cells exposed to PA compared to the positive control (only PA), while no effect was observed in cells treated only with cyanidin‐3‐*O*‐glucoside compared to the negative control (no PA) [52]. In a previous study, the same authors pretreated mature 3T3‐L1 with an extract rich in purified ACNs derived from bilberry and blackcurrant at a concentration of 20–40 µg/mL for 24 h, followed by treatment with PA at 1000 µM. The results showed an improvement in lipid accumulation in cells pretreated with ACNs compared to cells treated only with PA [50]. Mosqueda‐Solís et al. (2017) reported a reduction in lipid accumulation in 3T3‐L1 cells during the differentiation process following treatment with VA at 10 µM, but not at 1 µM [56]. Under our experimental conditions, the PPs tested failed to affect FFAs‐induced lipid accumulation in mature adipocytes. These conflicting findings may be dependent on the type and dose of PPs tested and/or the stage of adipocyte differentiation process. For example, Aranaz et al. (2019) tested several PPs, including FA and VA, at 10–100 µM under different conditions: pre‐, mid‐, and postdifferentiation of 3T3‐L1 cells for 8 days. The results have shown that flavonoids, FA, VA, and other phenolic acids at 100 µM, reduced lipid accumulation during the adipocytes differentiation process, while in mature adipocytes, the treatment with FA and VA, at 10–50 µM, failed to document a positive effect, in line with our observations [53]. Similarly, Hsu and Yen tested the effect of numerous phenolic acids (e.g., syringic, vanillic, ferulic, coumaric, caffeic, and chlorogenic) and flavonoids (naringenin, hesperidin, rutin, and quercetin) at 250 µM for 72 h during the differentiation of 3T3‐L1 cells, showing that only rutin and *o*‐coumaric acid reduced lipid accumulation while FA, SA, and VA, but other phenolics failed to demonstrate an effect on lipid accumulation [54].

Regarding the effect of WP PPs on glycerol release as a marker of lipolysis, we observed an increase in glycerol in the supernatant of the cell culture treated with FFAs alone and FFAs with WB PPs, while the NA treatment, as expected, significantly increased lipolysis compared to the other experimental conditions. The augmentation in lipolysis is enhanced by the FFAs treatments and the related increase in lipid storage. In various studies carried out in animals and humans, lipolysis increased in the adipose tissue due to the obesogenic environment, which caused insulin resistance, lipotoxicity, and adipocyte dysfunction [58, 59]. In our experiments, WB PPs did not affect glycerol release. This result seems in contrast with other studies reporting a positive effect on lipolysis following treatment with PA. For example, in the study of Kuppusamy et al., the treatment with FA at 10 µM for 24 h increased the level of glycerol in the supernatants of 3T3‐L1 cells after 8 days of differentiation compared to untreated cells [60]. Another study showed that the treatment with SA at 1000 µM for 48 h during the 3T3‐L1 differentiation significantly increased the cell medium glycerol [61]. Finally, in the study of Ziqubu et al., incubation with isoorientin at 10 µM for 4 h increased glycerol release compared to the control in mature 3T3‐L1 cells [62]. These conflicting findings may be attributed to the different experimental conditions adopted; in fact, while in our study WB PPs were tested along with FFAs, in the aforementioned studies, none tested PPs in the context of FFAs‐induced lipolysis.

To better elucidate the effect of WB PPs on lipid metabolism, several transcription genes involved in such modulation have been analyzed. One of the main key factors involved in lipogenesis is SREBP‐1c, a pivotal transcription factor that regulates lipid homeostasis at the level of adipocytes. It is considered the master regulator of adipogenesis and lipogenesis. In particular, it modulates genes involved in lipid synthesis, such as FASN, acetyl‐CoA carboxylase (ACC), and stearoyl‐CoA desaturase 1 (SCD‐1) in response to insulin and excess of energy [6, 63]. Other crucial transcription factors highly expressed in adipocytes are the PPAR‐γ, the liver X receptor (LXR), and the CCAAT‐element binding protein α (C/EBP‐α). Specifically, PPAR‐γ works as a fatty acids sensor, that in presence of lipids and FFAs, promotes other transcription factors such as LXR [64, 65]. The activation of LXR can induce the expression of SREBP‐1c, FASN, ACC, and FABP‐4 increasing lipogenesis [6, 66, 67]. However, overexpression of SREBP‐1c stimulates the generation of PPAR‐γ ligands, enhancing the activity of PPAR‐γ [7, 68]. Overall, this transcriptional network likely activates adipogenesis and lipogenesis with a positive feedback mechanism, increasing the expression of gene related to lipogenesis, including FABP‐4, FASN, adiponectin, and lipoprotein lipase (LPL) [7, 66, 69]. In particular, FABP‐4 is the main transporter involved in lipid uptake and metabolism, while FASN is the predominant enzyme involved in the synthesis of saturated fatty acids [7]. This complex of transcription factors orchestrates lipid metabolism in adipocytes, modulating the formation of new adipocytes, energy homeostasis, and insulin sensitivity [69].

In our experimental conditions, the treatment with FFAs at 750 µM did not affect the expression of SREBP‐1c and FABP‐4, while the level of FASN significantly increased, following FFA administration. At the same time, WB PPs did not influence the levels of SREBP‐1c and FABP‐4, while IA at 5 µM and VA at 0.5 and 5 µM significantly reduced the FFAs‐induced level of FASN, bringing it to the levels of the negative control.

Several studies reported the capacity of PPs to reduce markers of lipogenesis, including not only SREBP‐1c, FABP‐4, and FASN but also PPAR‐γ, C/EBP‐α, and ACC in 3T3‐L1 cells [50, 51, 52, 55, 57, 70, 71, 72, 73]. In contrast with our results, two studies reported that pretreatment with an extract of purified ACNs at 20–40 µg/mL or with cyanidin‐3‐*O*‐glucoside at 5–10 µM, followed by 24‐h exposure to PA (1000 µM) in mature 3T3‐L1 cells, reduced the gene expression of FABP‐4 and the protein production of PPAR‐γ, both induced by PA [50, 51]. However, cyanidin‐3‐*O*‐glucoside did not affect the level of FABP‐4 and PPAR‐γ compared to the negative control when tested without PA [51]. In a similar study, pretreatment with resveratrol at 200 µM reduced the gene expression of PPAR‐γ and SREBP‐1c enhanced by PA at 600 µM [70]. Although in the study of John and Arockiasamy, PA exposure at 100 µM during differentiation did not influence markers of lipid metabolism, including SREBP‐1c, PPAR‐γ, and FASN, treatment with SA, sinapic acid, hesperidin, and chrysin inhibited SREBP‐1c and FASN, while only SA reduced PPAR‐γ and C/EBP‐α [52]. In addition, several studies have shown that when 3T3‐L1 adipocytes were treated with phenolics or extracts rich in PPs after differentiation, the reduction of lipid accumulation was accompanied by a reduction of the lipogenic factors. For instance, exposure to FA at 100 µM for 48 h suppressed the expression of PPAR‐γ, C/EBP‐α, FASN, and ACC [55]. Moreover, VA at 1–10 µM for 48 h reduced the mRNA expression of PPAR‐γ, C/EBP‐α, and FABP‐4 [57]. Other studies evaluated the effect of PP‐rich extracts in mature 3T3‐L1 adipocytes. Kowalska et al. assessed the effect of two lingonberry extracts: one rich in ACNs, mainly containing cyanidin glycosides, and the other rich in PPs, containing derivatives from hydroxycinnamic acids, quercetin, and catechins. Both extracts, tested at 20 µg/mL for 24 h, decreased lipid accumulation and the level of FABP‐4 and FASN [71]. In another study, the authors tested an extract rich in quercetin derivatives, catechins, and gallic acid from blackberry nightshade at 300–500 µg/mL for 24 h in 3T3‐L1 cells. The findings revealed an inhibition in SREBP‐1c and FASN levels after the improvement of lipid storage [72]. In the study by Pérez‐Ramírez et al., an extract derived from azufrasin and Flor de Junio Dalia beans containing myricetin, kaempferol, quercetin derivatives, and catechin derivatives, at 150 µg/mL for 30 min in mature adipocytes, reduced the markers of lipogenesis, including PPAR‐γ, C/EBP‐α, FASN, and ACC [73].

The discrepancy between our study and those in the literature could be related to the different concentrations of PPs used. Most of the positive effects were obtained at supraphysiological and/or pharmacological concentrations. In our study, we used physiological concentrations, which might not be sufficient to improve lipid accumulation or lipolysis after 48 h in this model of mature 3T3‐L1 cells. One hypothesis to explain our results is that lipid accumulation influenced only FASN through a modulation mediated by PPAR‐γ and LXR, without the involvement of SREBP‐1c. Another explanation is that the production of SREBP‐1c and FABP‐4 reached a plateau due to the lipid droplets accumulated during the differentiation, preventing further expression despite FFAs treatment. Since PPs did not improve lipid accumulation, we expected similar results on the markers of lipogenesis, indeed SREBP‐1c and FABP‐4 remained unaltered. However, IA and VA were able to improve the production of FASN, although this effect did not translate in changes in lipid storage or lipolysis. We postulate that IA and VA exerted their effect through other factors involved in lipid metabolism, which were not analyzed in this study. These phenolics could directly or indirectly interact with other transcription factors such as PPAR‐γ, LXR, C/EBP‐α, or others that were not considered in our study. Additionally, since various studies have shown that lipid metabolism markers described above were modulated within 24 h, a kinetic evaluation of these markers over the 48‐h treatment period would be warranted.

Finally, regarding oxidative stress, our study found no significant effect on the levels of DNA damage, evaluated both as single‐strand breaks and 8OHdG level, and NRF2, upon treatment with WB PPs. This could imply that the physiological concentrations of PPs tested, or the times of exposure were insufficient to affect oxidative stress markers. Furthermore, we should also point out that, in our experimental conditions, the administration of FFAs did not induce oxidative stress conditions and this could partially explain the lack of an effect on the markers understudy. However, SA was able to counteract the reduction of HO‐1 caused by FFAs, indicating a specific antioxidant effect of this compound and a modulation of this marker. HO‐1 is known to play a crucial role in cellular defense against oxidative stress by degrading prooxidant heme into biliverdin, free iron, and carbon monoxide, which possess antioxidative properties [74]. Our findings are aligned with previous studies conducted when using phenolic acids. For instance, Zhang et al. reported that FA at 12.5, 25, and 50 µM significantly reduced both total ROS and mitochondrial superoxide production, in a dose‐dependent manner, 3T3‐L1 preadipocytes [75]. Also, Koh et al. demonstrated that the concentrations of 25, 50, and 100 µM FA were effective in positively modulating HO‐1 in 3T3‐L1, putatively through the activation of NRF2 and ERK pathways, highlighting the complexity of FA's role in oxidative stress regulation [55, 76]. In our study, the specific upregulation of HO‐1 by SA suggests a targeted antioxidative mechanism and a role of SA in mitigating oxidative stress‐induced damage in adipocytes, potentially through the modulation of the NRF2/HO‐1 pathway. Other studies have also documented the antioxidant effects of various phenolic compounds in adipocytes. For instance, Goya et al. reported that physiological concentrations (0.5, 1, 5, and 10 µM) of dihydrocaffeic, dihydroferulic, and hydroxyhippuric acids mitigated oxidative stress in adipocytes by upregulating antioxidant enzymes and reducing ROS levels in the 3T3‐L1 cell line [77]. Further, Vazquez Prieto et al. demonstrated that quercetin and catechin, at concentrations of 1 and 10 µM, individually or in combination, effectively reduced TNF‐α‐induced protein carbonylation in 3T3‐L1 adipocytes, which is a key indicator of oxidative stress [78]. These protective effects are likely attributed to their ability to modulate adipokine dysregulation through the inhibition of the MAPKs p38 and JNK, as well as the AP‐1 and PPAR‐γ signaling pathways. Although our WB PPs did not broadly modulate oxidative stress markers at the tested concentrations, the specific effect of SA on HO‐1 underscores the compound‐specific nature of phenolic interactions with oxidative stress pathways.

This study has several strengths that contribute to its biological relevance and potential translational value. Firstly, we employed physiologically relevant concentrations of blueberry‐derived metabolites, reflective of levels achievable through dietary intake, thereby enhancing the applicability of our findings. The use of FFA treatment to mimic an obesogenic environment provides a realistic in vitro model of lipid dysregulation. Additionally, we explored key pathways involved in lipid metabolism and oxidative stress, both of which are critically implicated in obesity‐related complications. This dual focus allowed for a more integrated understanding of the metabolic impact of the selected polyphenol metabolites.

However, we also acknowledge certain limitations. The use of murine‐derived, immortalized 3T3‐L1 adipocytes may not fully replicate human adipocyte physiology, and further validation in primary human cells or in vivo models would strengthen the translational significance. Although we investigated specific markers related to lipid metabolism and oxidative stress, other relevant pathways—such as lipolysis, glucose metabolism, and inflammation—were not addressed and warrant future study. Moreover, additional important blueberry metabolites not tested here, including hippuric acid and various Phase II conjugated derivatives (methylated, sulfated, and glucuronated compounds), warrant investigation for their potential biological effects. Finally, while we focused primarily on gene expression and protein levels for selected targets, expanding the analysis with broader transcriptomic or proteomic approaches could provide more comprehensive insights. Despite these limitations, our findings offer novel and meaningful contributions to the understanding of how dietary polyphenol metabolites may influence metabolic health, particularly in the context of obesity.

In conclusion, under our experimental conditions, VA and IA reduced the FFAs‐induced level of FASN, while SA seems to increase HO‐1 levels, which were otherwise reduced by FFAs treatment. The modulation of these enzymes is crucial for improving lipid metabolism and mitigating oxidative stress related to lipotoxicity in adipose tissue. These findings are encouraging since the modulation was achieved using WB PPs at concentrations similar to those found in the bloodstream after consuming a portion of fresh blueberries. Further studies are needed to corroborate these results by evaluating other metabolites from WB, including those from gut microbiota and phase two liver metabolism. Moreover, the analysis of other transcription factors pivotal in lipid metabolism and accumulation, such as PPAR‐γ and LXR, should be considered for future assessments. These analyses could enhance the overall understanding of WB PPs in the context of overweight and obesity prevention and management.

## Conflicts of Interest

The authors declare no conflicts of interest.

## Peer Review

The peer review history for this article is available at https://publons.com/publon/10.1002/mnfr.70101.

## Data Availability

The data used to support the findings of this study are included within the article.
